# Exploring the pharmacological mechanism of compound kushen injection in the treatment of breast cancer using *in vitro* experiments: Coupling network pharmacology with GEO database

**DOI:** 10.3389/fonc.2022.946758

**Published:** 2022-08-09

**Authors:** Yong Ye, Bo Zhang, Qiuyun Liang, Dandan Wang, Facheng Bai, Yuanhong Li, Lizhi Wei, Lilan Li, Huixue Huang, Yunxia Tang

**Affiliations:** ^1^ Pharmacy College, Guangxi Medical University, Nanning, China; ^2^ Scientific Research Center, Guilin Medical University, Guilin, China; ^3^ Pharmacy College, Guilin Medical University, Guilin, China; ^4^ Pharmacy Department, The First Affiliated Hospital of Guangxi Medical University, Nanning, China; ^5^ Pharmacy Department, The Second Affiliated Hospital of Guangxi Medical University, Nanning, China

**Keywords:** compound kushen injection, breast cancer, network pharmacology, gene expression omnibus, therapeutic mechanism

## Abstract

**Background:**

Breast cancer (BC) is one of the most common malignant tumors in women and poses a serious threat to their health. Compound Kushen injection (CKI) has shown therapeutic effects on a variety of cancers, including BC, and it can significantly improve the lives of patients. However, the underlying mechanism remains unclear and needs to be fully elucidated.

**Methods:**

The active constituents of CKI were identified through a literature review, and the anti-BC targets of CKI were determined using multiple databases and a ChIP data analysis. Subsequently, the target was analyzed on the DAVID database through GO and KEGG to identify the key pathway that CKI affects to exhibit anti-BC activity. In addition, MCF-7 and MDA-MB-231 cells were treated with CKI for 24 and 48 hours at five concentrations, and the effects of CKI on cell proliferation and apoptosis were measured using MTT and annexin V/propidium iodide staining assays, respectively. The genes and protein identified to be involved in this pathway were verified using real-time quantitative PCR (qPCR) and western blot(WB) in BC cells.

**Results:**

Twelve CKI anti-BC targets were obtained by a comprehensive analysis of the targets collected in the databases and results from the ChIP analysis. Bioinformatics analysis was performed for 12 targets. KEGG analysis showed that the 12 targets were mainly related to the VEGF, ErbB, and TNF signaling pathways. We focused our study on the VEGF signaling pathway as the *p*-value for the VEGF signaling pathway was the lowest among the three pathways. *In vitro* experiments showed that CKI significantly inhibited the proliferation of BC cells and induced apoptosis. Furthermore, qPCR and WB experiments showed that the expression of VEGF signaling pathway genes PIK3CA and NOS3 were significantly increased meanwhile SRC was significantly decreased after CKI intervention.

**Conclusion:**

CKI significantly inhibited the proliferation of BC cells and induced apoptosis. The main mechanism for the anti-BC effect of CKI may be that it regulates the VEGF signaling pathway by increasing the expression of PIK3CA, SRC, and NOS3. Macrozamin and lamprolobine may be the main active components of CKI against BC.

## Introduction

Breast cancer (BC) is a malignant tumor originating in mammary epithelial tissues. It is one of the most common malignant tumors in women and seriously threatens their health (1). The latest “Global Cancer Report 2020,” published by the World Health Organization showed that BC has become the most common cancer among women worldwide. In 2018, there were 2.1 million new cases of BC and 627,000 deaths (2). According to the 2020 Cancer Statistics published by the American Cancer Society in *CA: A Cancer Journal for Clinicians*, it was estimated that there would be 1,806,590 new cancer cases and 606,520 cancer deaths in 2020, ranking BC with the highest new incidence rate among female cancers. It is estimated that by 2030, the number of BC cases will reach 2.64 million, and the number of deaths may reach more than 1.7 million (3). Commonly used BC treatment methods include surgery, chemotherapy, radiation therapy, and endocrine therapy (4). However, even if early-stage BC patients are treated with the abovementioned treatments, 30%-40% of patients still experience recurrence and metastasis (5). In addition, chemotherapy often leads to different degrees of adverse reactions in patients, which further reduces its efficacy (6). Therefore, identifying effective drugs to treat BC remains an important task for drug researchers and doctors.

Compound Kushen injection (CKI) consists of a mixture of natural compounds extracted from *Radix Sophorae Flavescentis* and *Rhizoma Heterosmilacis* (mass ratio = 7:3). China has used CKI in combination with chemotherapy for the treatment of stomach, liver, and non-small cell lung cancers since 1995 (7). The main component of CKI is derived from *S*. *flavescens*, which contains matrine, oxymatrine, and other components with pharmacological activities. The extract from *S. flavescens* exhibits various pharmacological activities, including enhancing immunity, possessing anti-inflammatory, antiviral, and antiallergy properties, and providing cardiovascular protection; it also affects cancer significantly, such as inhibiting cancer cell proliferation, inducing cell cycle arrest, accelerating apoptosis, restraining angiogenesis, inducing cell differentiation, inhibiting cancer metastasis and invasion, reversing multidrug resistance, and preventing or reducing chemotherapy- and/or radiotherapy-induced toxicity when combined with chemotherapeutic drugs (8, 9). Clinical practice has shown that the use of CKI in chemotherapy treatments improved the functional status of and quality of life in patients after BC surgery and reduced adverse reactions (10). However, the mechanism of CKI in the treatment of BC remains unclear. CKI has multiple components and targets, which makes it difficult for traditional research methods to reveal its complex mechanism in the treatment of BC. Network pharmacology, with its characteristics of “multigene and multitarget” coincides with the complexity of traditional Chinese medicine therapy. Therefore, with the continuous development of systems biology in Chinese medicine research, network pharmacology has become a hot topic in the field of Chinese medicine (11).

**Figure 1:**
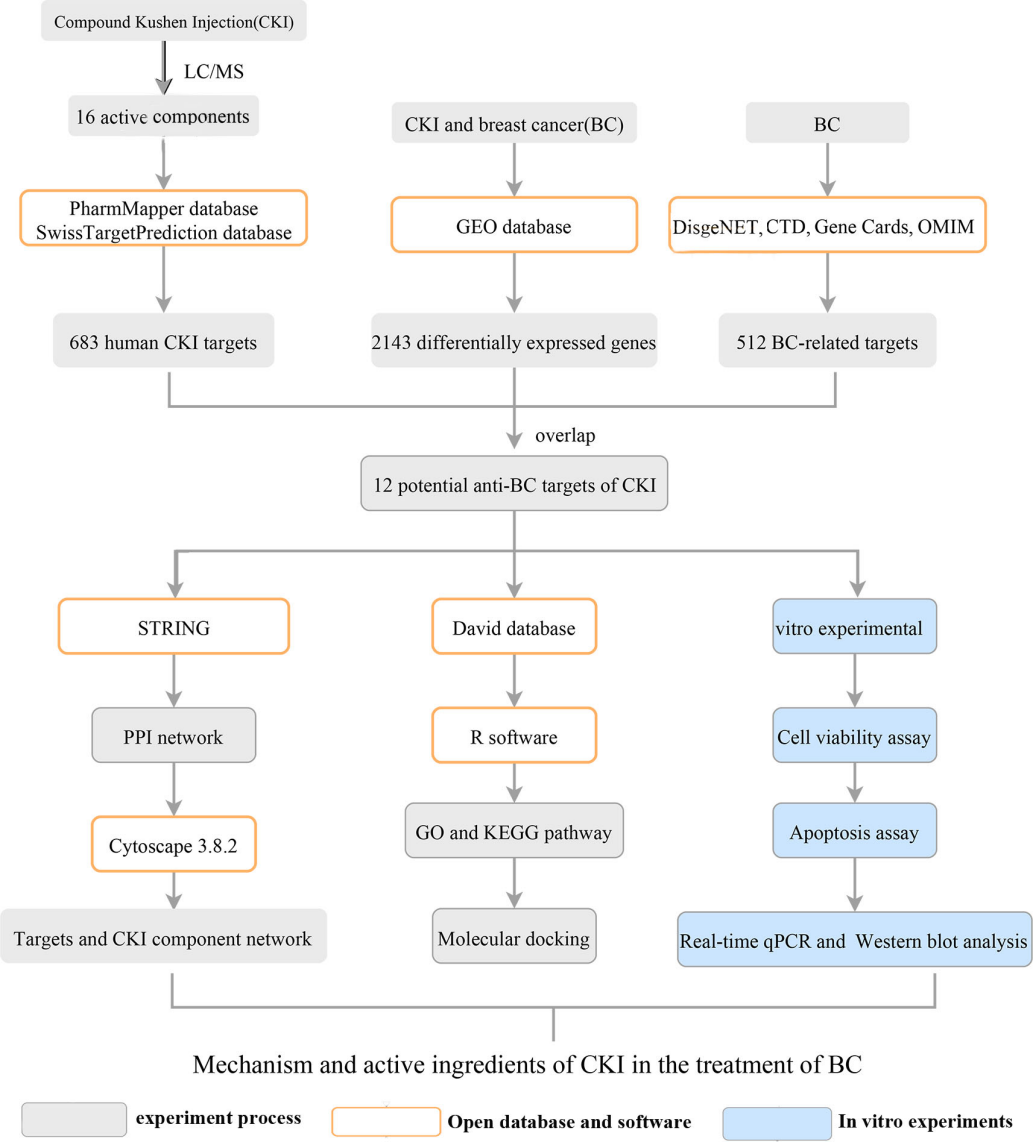
Flowchart of the study.

This study aimed to explore the mechanism of CKI in the treatment of BC using network pharmacology and Gene Expression Omnibus (GEO) data analysis and to verify the results using *in vitro* experiments. A scheme of the study protocol is shown in **Figure 1**.

## Materials and methods

### Collection of CKI active ingredients

Following literature search, CKI was analyzed by LC-MS/MS, and 21 active components were identified (12). After searching the PubChem database, 16 active components were selected for subsequent network pharmacology analysis (**Table 1**).

**Table 1:** Information on the active ingredients of CKI.

PubChem CID	Compound	Structure
15385684	9α-hydroxymatrine		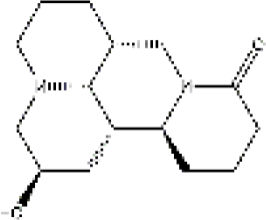
621307	baptifoline		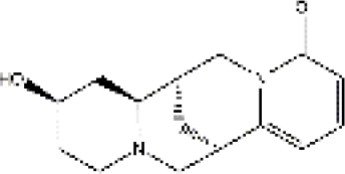
5271984	isomatrine		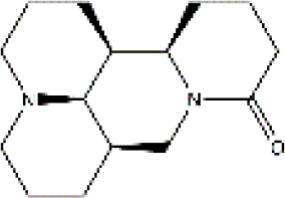
115269	sophocarpine		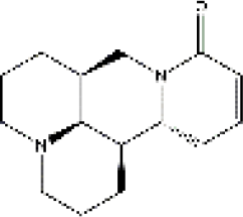
12442899	sophoranol		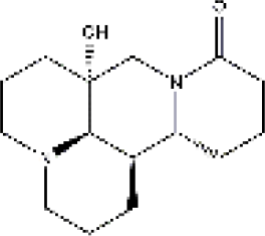
165549	sophoridine		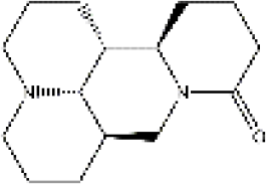
442827	trifolirhizin		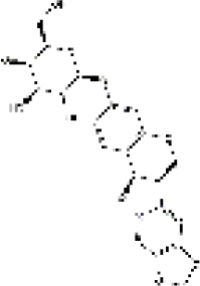
87752	lamprolobine		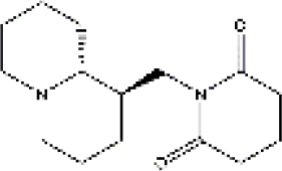
9576780	macrozamin		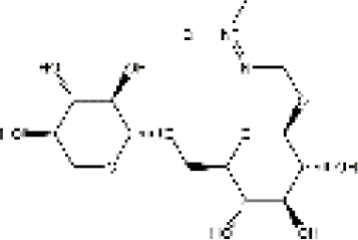
91466	matrine		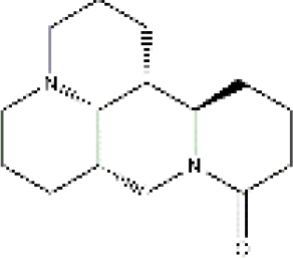
670971	N-methylcytisine		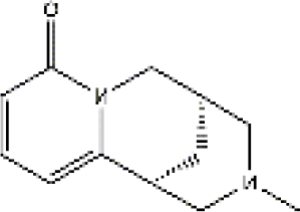
24864132	oxymatrine		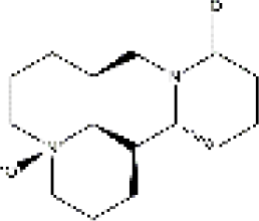
24721085	oxysophocarpine		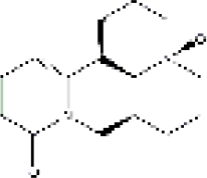
6710641	piscidic acid		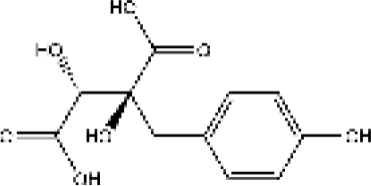
71773433	oxysophoridine		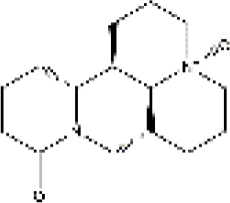
132555388	9α-hydroxysophocarpine		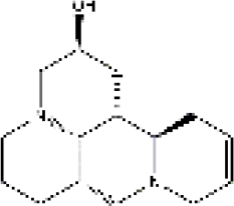

### Acquisition of CKI targets

The three-dimensional structures of the 16 active compounds were uploaded to the PharmMapper database (13–15) (http://www.lilab-ecust.cn/pharmmapper/). Based on the reverse pharmacophore matching method, the pharmacophore model of the drug was selected, and the final 100 protein conformations were set to obtain the target name, gene name, UniProt ID, and other results related to the 16 active compounds in CKI. Simultaneously, the active compounds of CKI were uploaded to the SwissTargetPrediction database (http://www.swisstargetprediction.ch/). The 16 targets identified from the database were combined and imported into the UniProt database (https://www.Uniprot.org/). Species “Homo sapiens” was selected, and repeated non-human and irregular targets were eliminated to obtain the protein targets related to CKI active components.

### Acquisition of BC targets

The targets of BC were searched for in the DisgeNET (https://www.disgenet.org/), CTD (http://ctd.mdibl.org/), Gene Cards (https://www.genecards.org/), OMIM (Https://omim.org/), and PharmGKB databases. To reduce false-positive results, the DisgeNET database selected genes with a score greater than 0.4. The CTD database selected genes with markers/mechanisms or therapeutic markers with an inference score greater than 100. The Gene Cards database selected genes scoring in the top 1%. The targets of the five databases were merged, and duplicate targets were removed. In addition, we searched the GEO database to find relevant ChIP data using “Compound Kushen Injection AND Breast Cancer” as the search phrase. The inclusion criteria were as follows: (1) the samples were human tissue or cells, and (2) a control group and a CKI treatment group were included. Subsequently, the raw data that met the criteria were downloaded and analyzed using HISAT2 and StringTie. The differential genes in the control and CKI treatment groups that overlapped with the genes identified from the abovementioned five database searches were deemed to be BC-related targets.

### Protein-protein interaction network construction

The intersection of CKI and BC-related proteins was used to obtain potential CKI anti-BC targets. The potential targets of anti-BC CKI were imported into the STRING (http://string-db.org/) database, the species was limited to humans, and the protein interaction relationship file of potential targets was obtained. Node1, node2, and the combined score were extracted from the protein interaction relationship file and imported into Cytoscape 3.8.2, to visualize the relationship between CKI components and targets.

### Bioinformatics analysis of CKI anti-BC targets

The protein names of selected CKI anti-BC targets were converted into gene symbols and imported into the David 6.8 database (https://david.ncifcrf.gov/) for GO and KEGG pathway enrichment analyses. The results were downloaded, and the data was sorted and visualized using the ggplot2 package in the R software.

### Data collection and analyses from TCGA database

Using TCGA database (http://gepia.cancer-pku.cn/), which includes 1085 BC tissues and 291 normal breast tissues (matched TCGA normal and GTEx data), mRNA expression of PIK3CA, SRC, and NOS3 in breast invasive carcinoma tissues and normal breast tissues was analyzed by Gene Expression Profiling Interactive Analysis (GEPIA).

### *In vitro* experimental verification of MCF-7 cells and MDA-MB-231 cells

#### Reagents

CKI was purchased from the Shanxi Zhendong Group. RPMI 1640 and Dulbecco’s modified Eagle’s medium (DMEM) were purchased from Gibco (USA). Thiazolyl blue tetrazolium bromide was purchased from Solarbio (China); Annexin V-FITC detection kit from Beyotime Biotechnology (China); fetal bovine serum from Gibco (USA); TRIzol reagent from Ambion (Carlsbad, CA, USA); and qPCR kit from TaKaRa (Japan). The PCR primers were purchased from Sangon Biotech Co., Ltd. (China). PIK3CA, SRC, NOS3 primary antibodies were purchased from Abclonal(Wuhan, China).

#### Cell viability assay

MCF-7 and MCF-10A were cultured in RPMI 1640, and MDA-MB-231 cells was cultured in Dulbecco’s modified Eagle’s media, containing 10% fetal bovine serum, 100 U/mL penicillin, and 0.1 mg/mL streptomycin at 37°C and 5% CO_2_. The cells were divided into 10 groups for each cell line. MCF-7 and MDA-MB-231 cells in the log phase were seeded into 96-well plates at a density of 2×10^4^ cells/well. After 24 hours, cells were introduced to media containing CKI (calculated based on the 20.8 mg/mL total alkaloids of CKI)—MCF-7 cells were cultured with 0, 2, 4, 6, and 8 mg/mL, and MDA-MB-231 cells were cultured with 0, 1, 2, 4, and 8 mg/mL of CKI. Each group contained six parallel replicates. The culture was continued for 24 hours and 48 hours, upon which the media was exchanged for one containing MTT. After a 4-hour incubation at 37°C, the supernatant was discarded, and DMSO (100 µL) was added to each well to dissolve the formazan. The plates were then shaken for 10 minutes before the absorbance (OD) was measured at 490 nm using a microplate reader to calculate the survival rate.

#### Apoptosis assay

MCF-7, MCF-10A and MDA-MB-231 cells were cultured in 6-well trays and treated with a solution containing 2 mg/mL CKI. After 48 hours of treatment, the cells were harvested, and the rate of apoptosis was measured using an Annexin V-FITC detection kit according to the manufacturer’s instructions. The stained cells were sorted and data acquired on an BD FACSCanto Plus (BD Biosciences, NJ, USA) and the data were analysed using FlowJo software V10 (TreeStar Inc.,OR, USA).

#### Real-time qPCR

MCF-7 and MDA-MB-231 cells were treated with a solution containing 2 mg/mL CKI. After 48 hours of treatment, the media were discarded and the cells washed with PBS for five times. Total RNA from each group was extracted using the TRIzol reagent, and the absorbance was measured at 260 nm to determine the total RNA concentration. Random hexamer primers and a SuperScript III reverse transcriptase kit were used to obtain complementary DNA (cDNA). After reverse transcription, a 20-μL aliquot was used for qPCR to detect the relative expression of mRNA. The results were analyzed using the 2^-ΔΔCt^ method. The primer sequences are tabulated in **Table 2**.

**Table 2:** Primer Sequences.

Target Gene	Forward primer (5’- 3’)	Reverse primer (5’- 3’)
PIK3CA	TCTGTCTCCTCTAAACCCTG	TTCTCCCAATTCAACCAC
NOS3	AGCTGCCCTGATGGAGATGT	CCCGAACACACAGAACCTGAG
SRC	GCGAGAAAGTGAGACCACGA	CCATCGGCGTGTTTGGAGTA
Actin	TGGCACCCAGCACAATGAA	CTAAGTCATAGTCCGCCTAGAAGCA

#### Western blot analysis

MCF-7 and MDA-MB-231 cells were treated with a solution containing 2 mg/mL CKI. After 48 hours of treatment, the total protein were extracted from the MCF-7 and MDA-MB-231 cells, respectively. The bicinchoninic acid (BCA) protein assay kit was used to analyze the concentrations for total protein according to the manufacturer’s protocol. Protein from MCF-7 and MDA-MB 231 cells was isolated using SDS-PAGE with a 10% separating gel by loading 20μg of protein per lane. Then, the proteins were transferred onto a PVDF membrane. Afterward, the membranes were blocked with 5% fat-free milk in TBST buffer for 40min, followed by incubation with different primary antibodies including PIK3CA, SRC, NOS3 and β-actin overnight at 4°C. The next day, PVDF membranes were washed three times in TBST buffer and incubated with an anti-rabbit secondary antibody for 50min at 37°C. Finally, the relative expressions of various proteins were quantified by Image-J. The grey densities of the protein bands were normalized by using β-actin density as an internal control.

### Molecular docking

Molecular docking was performed using SYBYL-X 2.0 software (Tripos, St. Louis, MO, USA). The structures of the 12 proteins listed in **Table 1** used for molecular docking were searched from the UniProt database (https://www.uniprot.org/). For the protein preparation, eutectic ligands and water molecules contained in the protein structure were removed, residues were repaired, side chains were fixed, and hydrogenation was performed. In parallel, small molecules related to the 12 targets were minimized in energy, the force field was set, and the algorithm was optimized. Finally, Surflex-Dock in SYBYL-X 2.0 was used to dock individual small molecules into the corresponding protein crystal structures. The binding mode with a C_Score ≥ 4 and total score ≥ 5 was selected for subsequent analysis; PyMol and LigPlot were used to visualize the results.

### Statistical analysis

SPSS 20.0 was used to perform statistical analysis. Differences between the groups were assessed using a Student’s t-test. The data are presented as mean ± SE, and comparisons with a *p*-value < 0.05 were considered to be statistically significant.

## Results

### Anti-BC targets of CKI analysis

**Figure 2:**
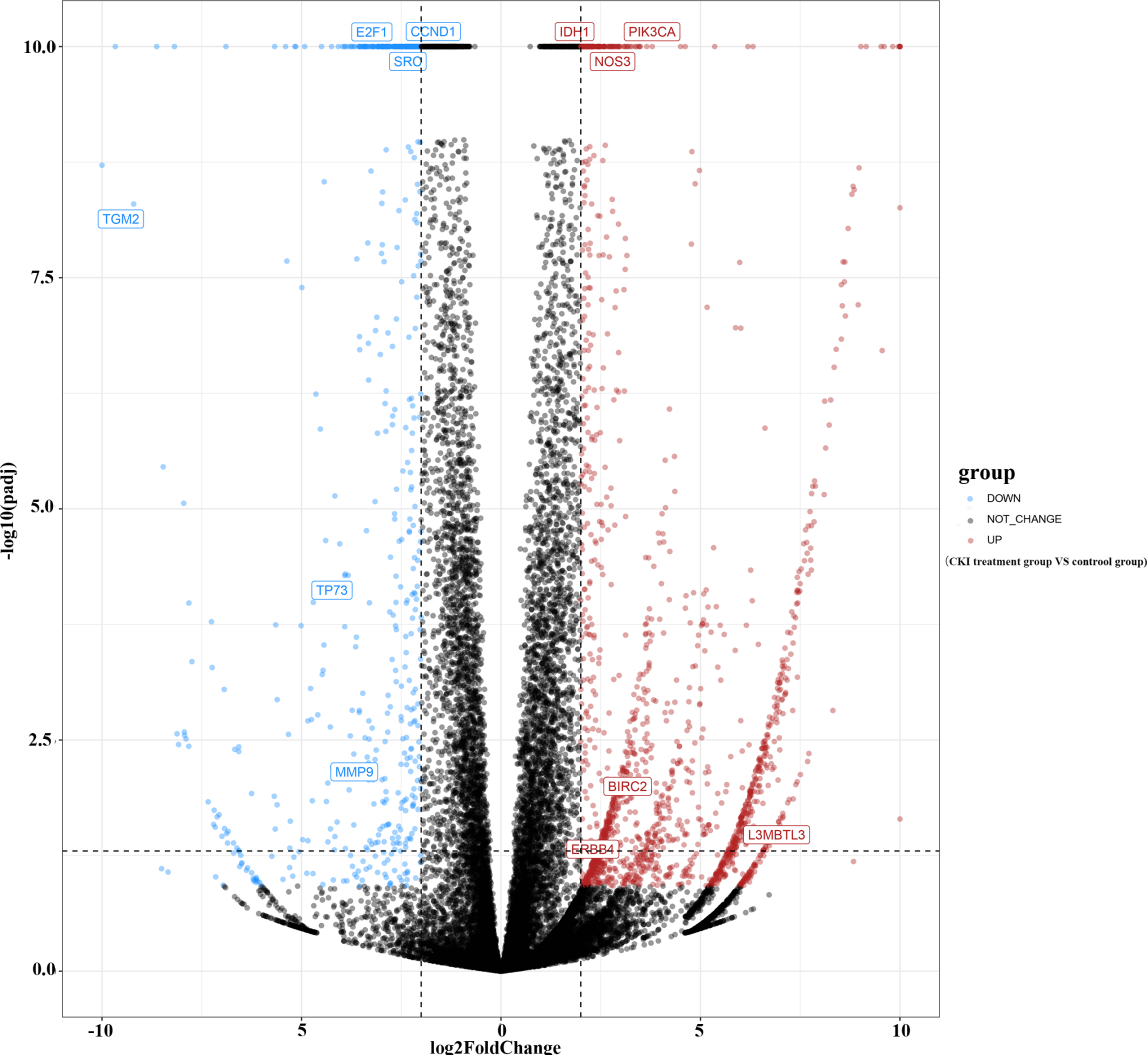
Gene difference analysis of chip GSE78512. Notes: The –log10(padj) was set equal to 10 when it was greater than 10.

**Figure 3:**
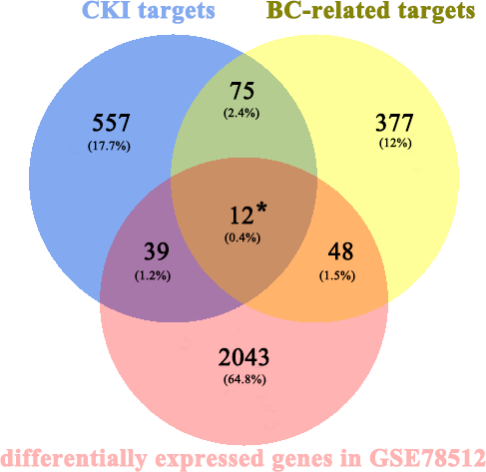
Venn diagram for CKI prediction of target genes and disease-related genes. * represent the potential anti-BC targets of CKI.

A total of 683 human CKI targets were collected from the PharmMapper and SwissTargetPrediction databases. Additionally, the results obtained from the DisgeNET, CTD, Gene Cards, OMIM, and PharmGKB databases showed that there was a total of 512 BC-related targets. Subsequently, according to the ChIP analysis inclusion criteria, one chip meeting the criteria was screened: GSE78512. In this data set, we selected the MCF-7 control group and the treatment group with the most pronounced effect, which was the group treated with 2 mg/mL CKI for 48 hours, for the follow-up study. As the mRNA expression was not analyzed in the literature, raw data were analyzed. Raw data counts were exported for a gene difference analysis. The results showed 2143 differentially expressed genes between the MCF-7 control group and CKI treatment group (**Figure 2**). Finally, we took the intersection of CKI targets, BC-related targets and differentially expressed genes in GSE78512. Then, 12 potential anti-BC targets of CKI were found **(** **Figure 3** **)**.

### Targets and CKI component network

**Figure 4:**
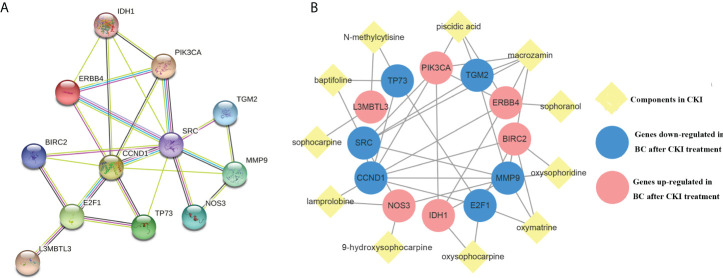
Protein-CKI active ingredients interaction network. Notes: **(A)** Protein-protein interaction (PPI) network of 12 CKI anti-BC targets. **(B)** CKI active ingredients- anti-BC targets interaction network.

The STRING database was used to generate data for 12 target-related protein-protein interaction(PPI) network (**Figure 4A**). Subsequently, interaction relationships among the 12 targets and those with the CKI active components were imported into Cytoscape to construct a functional-related protein interaction network (**Figure 4B**).

### Biological function and pathway enrichment analyses

**Figure 5:**
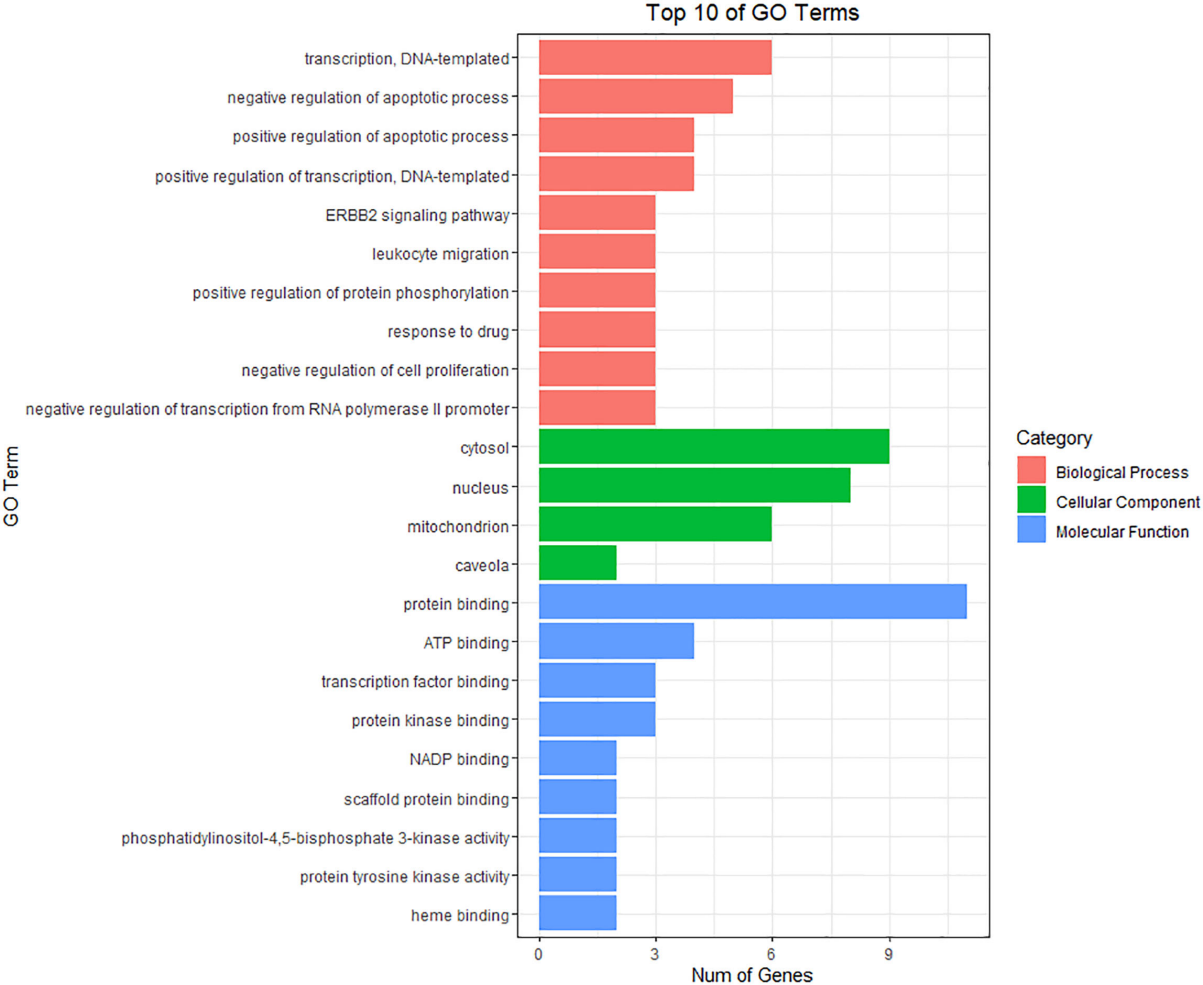
GO enrichment analysis for 12 CKI anti-BC targets.

**Figure 6:**
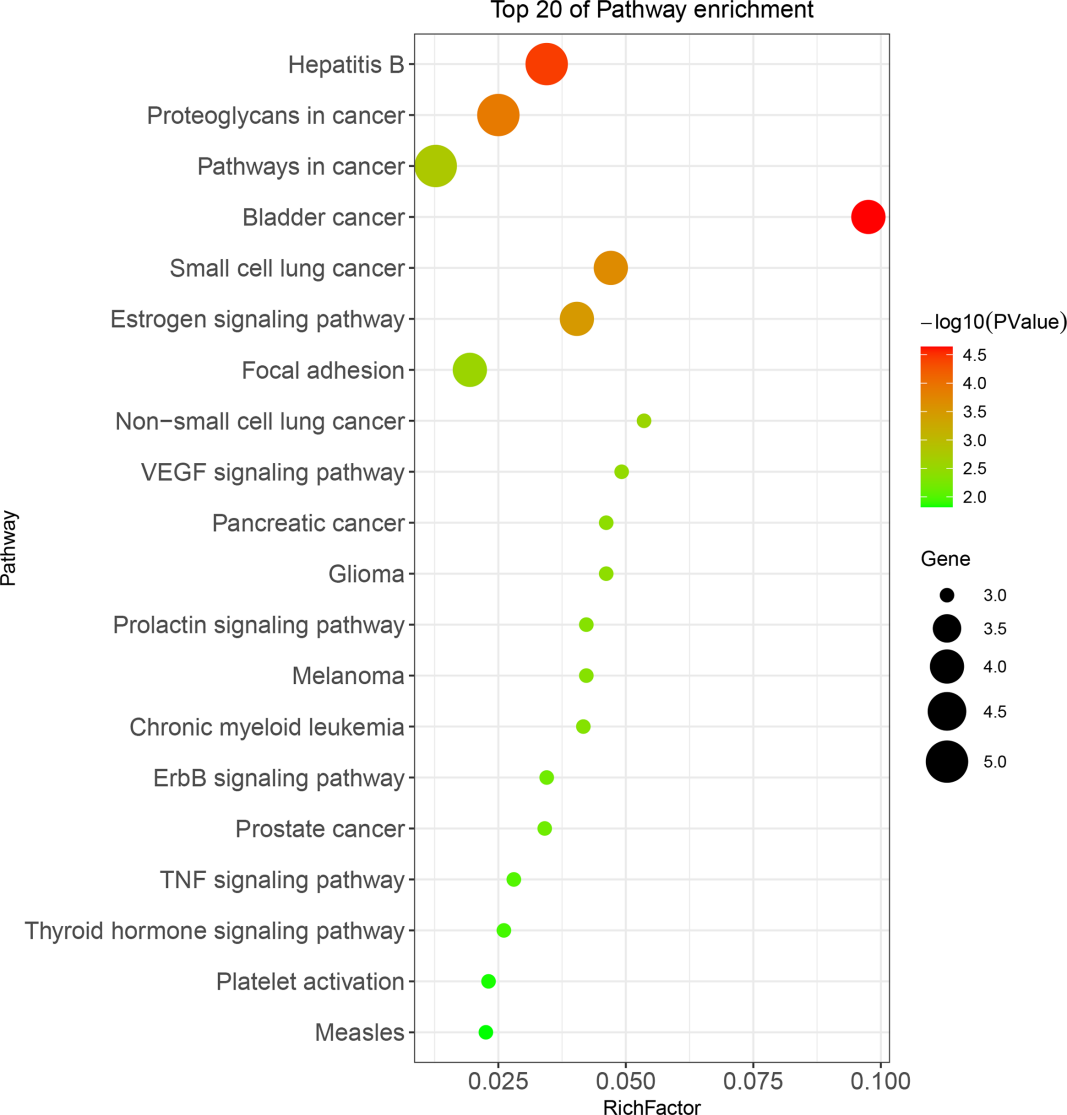
KEGG pathway analysis for 12 CKI anti-BC targets.

The anti-BC targets of CKI were imported into the David database for Gene Ontology(GO) and KEGG analyses. The results showed that the 12 targets were involved in 43 biological processes (BP), 4 cell components (CC), and 9 molecular functions (MF), including, but not limited to, events related to transcription (DNA-templated) and regulation of the apoptotic process, actions occurring in the cytosol, mitochondria, and nucleus, and activities associated with protein, ATP, and transcription factor binding. The top 10 items involved in each gene function were selected and drawn into a bar graph using R 4.10 software (**Figure 5**). In addition, enrichment analysis results of the KEGG pathway showed that 12 targets were involved in regulating 30 signal pathways; 20 signal pathways with minimum *p*-values were selected to draw bubble maps (**Figure 6**). The vascular endothelial growth factor (VEGF), epidermal growth factor receptor (ErbB), and tumor necrosis factor (TNF) signaling pathways are the three main pathways related to BC.

### The mRNA expression of PIK3CA, SRC, and NOS3 in BC from The Cancer Genome Atlas (TCGA) data and chip GSE78512

**Figure 7:**
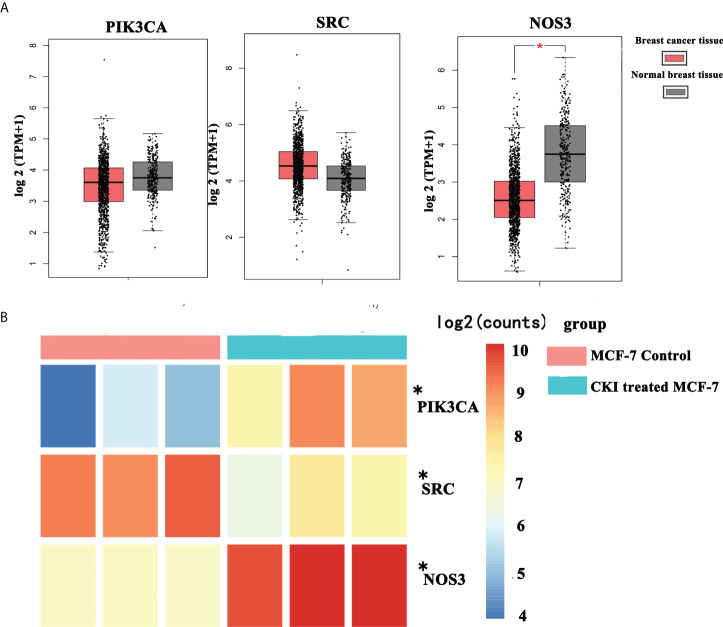
The mRNA expression of PIK3CA, SRC and NOS3 in breast cancer from TCGA data and chip GSE78512. Notes: **(A)** The mRNA expression of PIK3CA, SRC and NOS3 in breast cancer from TCGA data. **(B)** The mRNA expression of PIK3CA, SRC and NOS3 in breast cancer from chip GSE78512.*P < 0.05, compared with normal breast tissue group or MCF-7 control group. Thanks again for your meticulous review.

Among the signaling pathways related to BC in enrichment analysis results of the KEGG pathway, the VEGF signaling pathway, which had the smallest *p-*value (*P*=0.0023), was selected for an in-depth study. The KEGG analysis showed that the genes involved in the regulation of the VEGF signaling pathway in anti-BC targets of CKI were PIK3CA, SRC, and NOS3. Subsequently, data mining on TCGA database found that BC tissues showed decreased PIK3CA and increased SRC expression compared to the expression of these genes in normal tissues(*P*>0.05); in addition, the BC tissues showed decreased significantly NOS3 compared to the expression of these genes in normal tissues(*P*<0.05) (**Figure 7A**). The results of the chip GSE78512 analysis showed that after CKI treatment, the expression of PIK3CA and NOS3 increased and the expression of SRC decreased in MCF-7 cells (**Figure 7B**). Comparing the expression of PIK3CA, SRC, and NOS3 in normal breast tissue and breast cancer tissue in TCGA database, it can be found that after CKI treatment, the expression of PIK3CA, SRC, and NOS3 in BC cells tends to be in normal tissue, suggesting that CKI may regulate the VEGF signaling pathway by reversing the abnormal expression of PIK3CA, SRC, and NOS3.

### CKI inhibits MCF-7 and MDA-MB-231 proliferation and induces apoptosis

**Figure 8:**
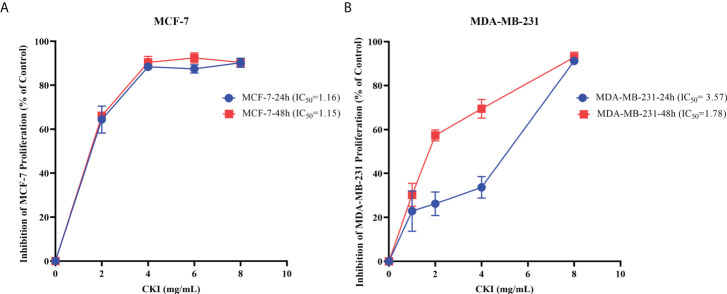
Inhibition of MCF-7 and MDA-MB-231 proliferation. Notes: **(A)** The inhibition of MCF-7 proliferation (% of Control). **(B)** The inhibition of MDA-MB-231 proliferation (% of Control). n=3. Data are presented as means ± SD.

**Figure 9:**
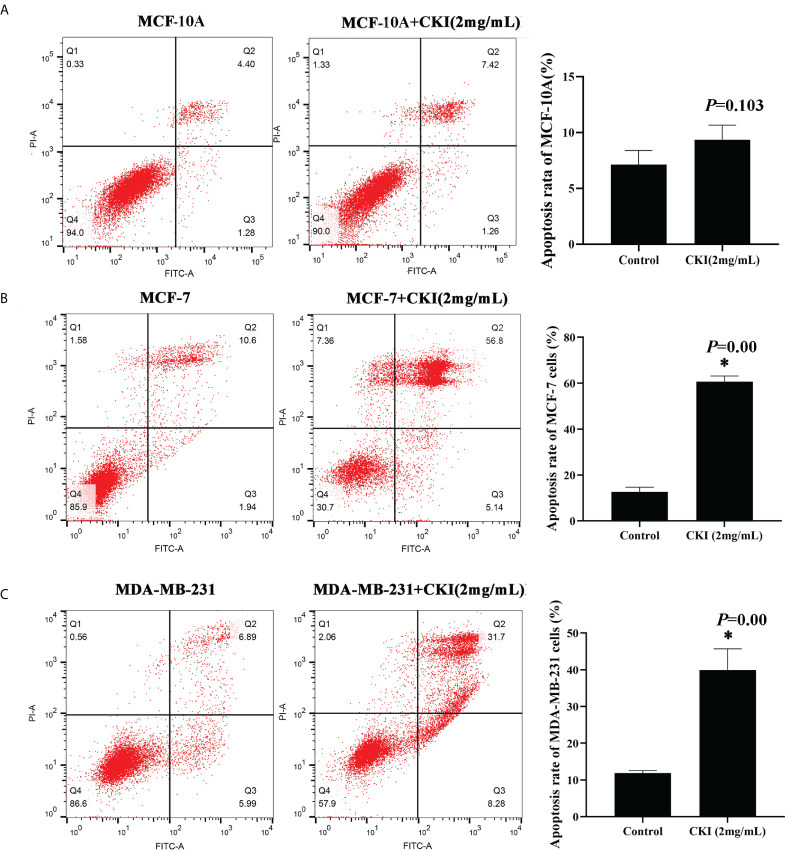
CKI induces apoptosis of MCF-7 cells and MDA-MB-231 cells. Notes: **(A)** Induction of apoptosis in MCF-10A cells with CKI treatment. **(B)** Induction of apoptosis in MCF-7 cells with CKI treatment. **(C)** Induction of apoptosis in MDA-MB-231 cells with CKI treatment. The level of apoptosis was determined by measuring the levels of Annexin V-FITC and PI staining. n=3. Data are presented as means ± SD. *P < 0.05, compared with MCF-10A(MCF-7 or MDA-MB-231) group.

To explore the effect of CKI on the proliferation of MCF-7 and MDA-MB-231 cells, we used an MTT assay to measure cell viability after treatment with various doses of CKI. The proliferation of MCF-7 and MDA-MB-231 cells were significantly inhibited, and the IC_50_ of CKI for MCF-7 and MDA-MB-231 cells after 24 hours (48 hours) of treatment was 1.16 (1.15) mg/mL and 3.57 (1.78) mg/mL, respectively (**Figure 8**). An annexin V/propidium iodide assay was used to quantify apoptosis when MCF-10A, MCF-7 and MDA-MB-231 cells were treated with CKI. Compared to untreated cells, the percentage of apoptotic cells did not change significantly in MCF-10A; however, the percentage of apoptotic cells increased in MCF-7 and MDA-MB-231 cells treated with CKI, indicating that CKI-treated BC induced apoptosis without affecting normal cells (**Figure 9**).

### The mRNA and protein expression of PIK3CA, SRC, and NOS3 in MCF-7 and MDA-MB-231 cells

**Figure 10:**
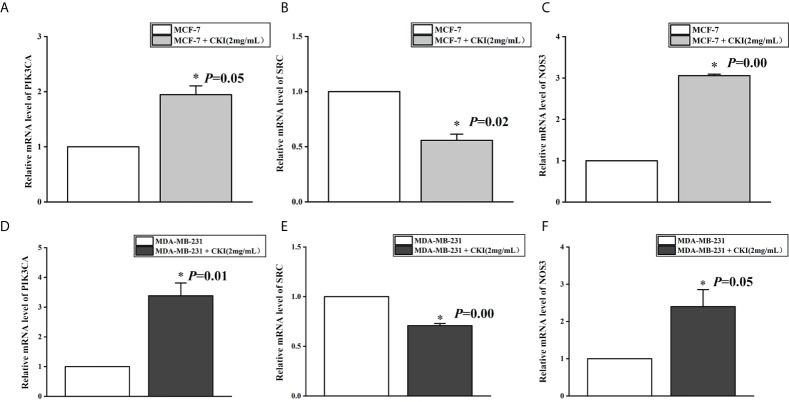
The mRNA expression of PIK3CA, SRC and NOS3 in MCF-7 cells and MDA-MB-231 cells. Notes: **(A)** The expression levels of PIK3CA mRNA in MCF-7 cells; **(B)** The expression levels of SRC mRNA in MCF-7 cells; **(C)** The expression levels of NOS3 mRNA in MCF-7 cells; **(D)** The expression levels of PIK3CA mRNA in MDA-MB-231 cells; **(E)** The expression levels of SRC mRNA in MDA-MB-231 cells; **(F)** The expression levels of NOS3 mRNA in MDA-MB-231 cells. n=3. Data are presented as means ± SD. *P < 0.05, compared with MCF-7(MDA-MB-231) group.

**Figure 11:**
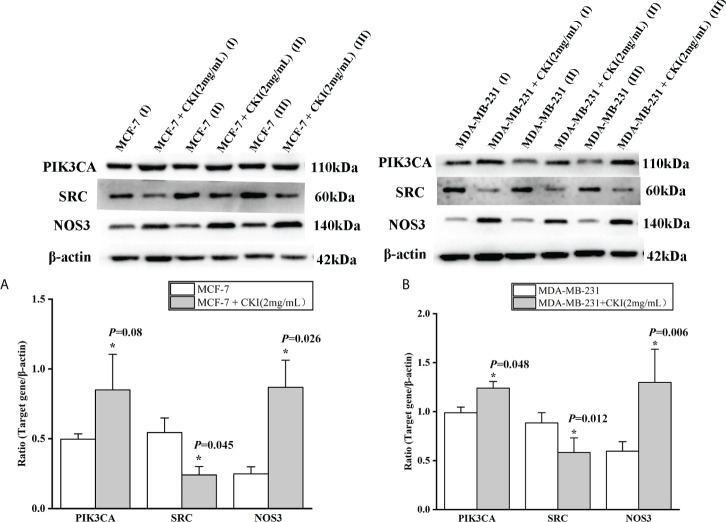
The protein expression of PIK3CA, SRC and NOS3 in MCF-7 cells and MDA-MB-231 cells. Notes: **(A)** The expression levels of PIK3CA, SRC and NOS3 protein in MCF-7 cells; **(B)** The expression levels of PIK3CA, SRC and NOS3 protein in MDA-MB-231 cells. Lane-I, III, V represented MCF-7 (MDA-MB-231) cells group; Lane-II, IV, V represented MCF-7 (MDA-MB-231) cells with 2 mg/ml CKI treatment group. n=3. Data are presented as means ± SD. *P < 0.05, compared with MCF-7(MDA-MB-231) group.

To provide further evidence, we performed the same *in vitro* experiments as for chip GSE78512. The experiments were performed with MDA-MB-231 cells as well to further corroborate our conclusion. In addition, we also carried out corresponding experiments at the protein level.The results showed that the expression of PIK3CA and NOS3 mRNA and protein in MCF-7 and MDA-MB-231 cells were significantly increased and the expression of SRC mRNA were significantly decreased after treatment with CKI (**Figures 10**, **11**). Since PIK3CA, SRC, and NOS3 were enriched in the VEGF signaling pathway, our results indicated that CKI may regulate VEGF signaling pathway in the treatment of BC.

### Molecular docking

**Figure 12:**
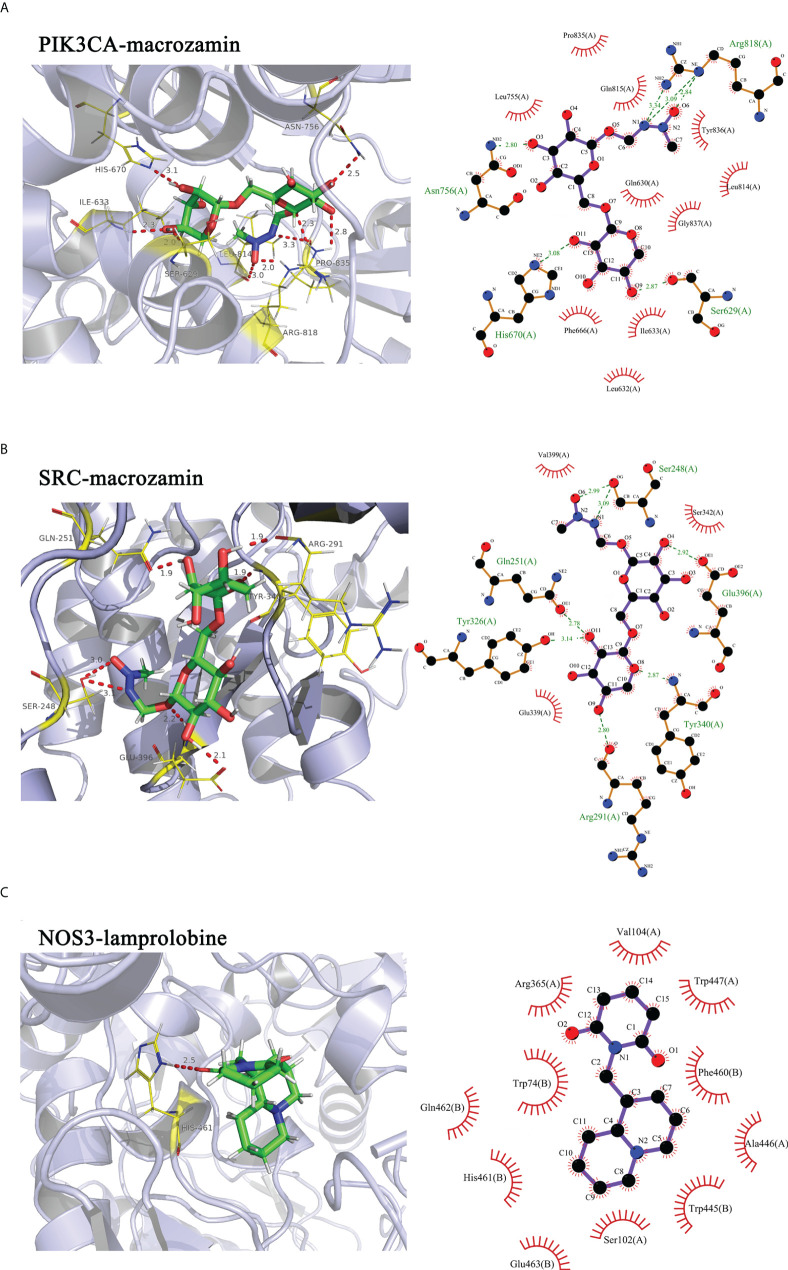
Molecular docking simulations of the interactions between the active components of CKI and the predicted targets. Notes: **(A)** PIK3CA-macrozamin; **(B)** SRC-macrpzamin; **(C)** NOS3-lamprolobine.

To further elucidate the possible interactions between the active components of CKI and predicted targets related to the VEGF signaling pathway, we performed molecular docking simulations of the interactions between the active components of CKI and the predicted targets. The results showed that PIK3CA, SRC, and NOS3 interact well with the active small molecules in CKI; the docking scores are tabulated in **Table 3**. PIK3CA binds to macrozamin possibly by interacting with the residues His-670, Ser-629, Asn-756, and Arg-818 through hydrogen bonds and the residues Gln-630, Leu-632, Ile-633, Phe-666, Leu-755, Leu-814, Gln-815, Pro-835, Tyr-836, and Gly-837 *via* hydrophobic interactions (**Figure 12A**). SRC binds to macrozamin possibly by interacting with the residues Ser-248, Gln-251, Arg-291, Tyr-326, Tyr-340, and Glu-396 through hydrogen bonds and with the residues Glu-339, Ser-342, and Val-399 *via* hydrophobic interactions (**Figure 12B**). NOS3 binds to lamprolobine possibly by interacting with the residue His-461 through hydrogen bonds, and the residues Ser-102, Arg-365, His-461, Gln-462, Glu-463, Trp-74, Val-104, Trp-445, Ala-446, Trp-447, and Phe-460 *via* hydrophobic effects (**Figure 12C**). The docking results further suggest that the VEGF signaling pathway may be involved in CKI therapy for BC.

**Table 3:** Docking information of VEGF signaling pathway-related predicted target with active components of CKI.

gene	PDB ID	PDB Entry	Compound	Total Score	Crash	Polar	C Score
NOS3	P29474	4D1P	lamprolobine	5.3214	-2.4285	0.5205	5
NOS3	P29474	4D1P	9α-hydroxysophocarpine	4.4065	-0.5009	2.4408	2
PIK3CA	P42336	4JPS	macrozamin	7.1966	-1.3812	5.9772	4
PIK3CA	P42336	4JPS	baptifoline	4.4568	-0.9861	0	5
SRC	P12931	2H8H	macrozamin	8.0547	-1.4214	8.6387	5
SRC	P12931	2H8H	piscidic acid	4.6232	-1.3682	4.6074	3

## Discussion

This study explored the mechanism of CKI in the treatment of BC through network pharmacology and GEO database analysis combined with *in vitro* experimentation. First, we collected 683 CKI and 512 BC-related targets through multiple database analyses. To avoid false-positive results, we searched the GEO database for chips related to CKI treatment of BC to verify the collected targets. According to our research criteria for chip selection, one chip was found: GSE78512. After analyzing GSE78512, 2143 differentially expressed genes were identified, and 12 CKI anti-BC targets were obtained after comprehensive analysis with targets collected from the database. Bioinformatics analysis was then performed for 12 targets. GO analysis showed that these 12 targets were mainly related to apoptosis and cell proliferation. KEGG analysis showed that the 12 targets were mainly related to the VEGF, ErbB, and TNF signaling pathways. Among them, the VEGF signaling pathway had the lowest *p*-value, which became the focus of our attention.

VEGF is a mitogen that is specific to endothelial cells. Its structure is a homologous dimer glycoprotein linked through disulfide bonds that can induce physiological and pathological angiogenesis (16). Studies have shown that VEGF binds to VEGFR2 on the surface of endothelial cells and triggers a cascade of signal transduction *via* the PI3K and mitogen-activated protease (MAPK) pathways. These pathways, involving key messengers nitric oxide and cyclic cGMP, contribute to endothelial mitosis, promote endothelial cell survival through the apoptotic inhibitor protein pathway, and promote endothelial proliferation, leading to angiogenesis (17, 18). Additionally, VEGF effectively increases the permeability of endothelial cells and enhances the ability of tumor cells to metastasize (19). Many studies on the inhibition of BC proliferation and metastasis and the mechanisms of action described are related to the VEGF signaling pathway. Wang et al. found that cystathionine-γ-lyase can promote BC metastasis by regulating the VEGF signaling pathway (20). Lee et al. studied the role of VEGF in brain metastases originating from BC. Human brain microvascular endothelial cells were used to build an *in vitro* model of blood vessels, and using VEGF inhibitors, BC metastasis to the brain was suppressed (21). Ghattass et al. used drugs targeting hypoxia-inducible factor-1α *in vivo* and *in vitro* to demonstrate their antimetastatic effect on BC and verified that the mechanism of drug-induced cell damage was related to the downregulation of VEGF expression (22). In addition, Tian et al. showed that asiatic acid could inhibit angiogenesis and vascular permeability by acting on the VEGF/VEGFR2 signaling pathway, thereby inhibiting the growth and metastasis of BC in mice (23). In this study, we compared the relative expression of CKI anti-BC targets involved in the VEGF signaling pathway in BC and normal tissues from the TCGA database. Then, through the chip GSE78512 raw data analysis, it was found that CKI can reverse the expression of the anti-BC targets in BC cells. Furthermore, through *in vitro* experiments, we showed that CKI can inhibit proliferation and induce apoptosis of BC cells. These results suggest that CKI may exert its anti-BC effect by regulating the VEGF signaling pathway to inhibit proliferation and induce apoptosis in BC cells.

Molecular docking is an established in silico structure-based method widely used in drug discovery. Docking enables the identification of novel compounds of therapeutic interest and predicts ligand-target interactions at the molecular level (24). To further provide evidence that the mechanism of CKI treatment in BC was related to the VEGF signaling pathway, we carried out molecular docking experiments between the active components of CKI and the targets (PIK3CA, SRC, and NOS3) involved in the VEGF signaling pathway.

PIK3CA gene was detected by Volinia et al. In 1994 using *in situ* hybridization (25), and it is the only gene in the PI3K family that can undergo somatic mutation and cause cancer. PIK3CA gene mutation leads to the growth and transformation of mammary epithelial cells and fibroblasts, and inhibits apoptosis, which is closely related to the occurrence and development of tumors (26, 27). About 20-40% of breast cancers contain PIK3CA mutations, and the frequency of PIK3CA mutations is second only to TP53 mutations in breast cancer (28). Therefore, regulating the expression of PIK3CA may help improve breast cancer development. SRC family kinases are group of nine non-receptor tyrosine kinases of which SRC is the prototype (29). A key regulator of cell-matrix and cell-cell adhesions, SRC has been implicated in a number of cancer-associated phenotypes, including proliferation, migration and invasion (30, 31). Disease-associated upregulation of SRC kinase activity has been demonstrated in many malignancies, including breast, colon and gastric cancer. In breast cancer, elevated SRC expression is associated with poor prognosis (32). In addition, members of the SRC family may phosphorylate NOS3, leading to a decrease in NOS3 activity (33, 34). Studies have shown that NOS3 may play an important role in tumor progression *via* angiogenesis or apoptosis (35). Therefore, SRC and NOS3 play an important role in the progression of breast cancer.

In our study, the results showed that PIK3CA-macrozamin, SRC-macrpzamin, and NOS3-lamprolobine had favorable binding energies, further indicating that CKI may treat BC by regulating the VEGF signaling pathway.

Unfortunately, although we found through network pharmacology and GEO database that CKI anti-BC may regulate VEGF signaling pathway, and proved that gene/protein expression levels enriched in VEGF signaling pathway could be reversed by CKI treatment at nucleic acid and protein levels. However, whether CKI has an effect on normal endothelial cells is lacking in this study.

## Conclusion

In summary, CKI could inhibit proliferation and induce apoptosis of BC cells, and the therapeutic mechanism of CKI against BC is likely to be closely related to its regulation of the VEGF signaling pathway by reversing the expression of PIK3CA, SRC, and NOS3. In addition, network pharmacology may be used to predict key therapeutic targets. We found that macrozamin and lamprolobine may be the active compounds in CKI that act against BC.

## Data availability statement

Publicly available datasets were analyzed in this study. This data can be found here: GEO:GSE78512.

## Author contributions

YY completed most of the research and drafted manuscripts. BZ, QL, and DW plotted all of the charts. YT, YL, and LW analyzed the data. LL did the experiment *in vitro*. HH and FB designed the research and reviewed the manuscript. All authors contributed to the article and approved the submitted version.

## Funding

Our study was supported by National Natural Science Foundation of China (No.81960756, No.81360689), Guangxi natural science foundation (No.2022GXNSFDA035063, No.2018GXNSFAA050078), Innovation and Entrepreneurship Training Program for College Students (202110601023) and Guangxi Zhuang Autonomous Region Health and Family Planning Commission Self-financed Scientific Research Project (Z20211128, Z20210927).

## Conflict of interest

The authors declare that the research was conducted in the absence of any commercial or financial relationships that could be construed as a potential conflict of interest.

## Publisher’s note

All claims expressed in this article are solely those of the authors and do not necessarily represent those of their affiliated organizations, or those of the publisher, the editors and the reviewers. Any product that may be evaluated in this article, or claim that may be made by its manufacturer, is not guaranteed or endorsed by the publisher.

## Abbreviations

BC, breast cancer; CKI, Compound Kushen injection; ErbB, epidermal growth factor receptor; GEO, Gene Expression Omnibus; GEPIA, Gene Expression Profiling Interactive Analysis; GO, Gene Ontology; KEGG, Kyoto Encyclopedia of Genes and Genomes; PPI, protein-protein interaction; TCGA, The Cancer Genome Atlas; TNF, tumor necrosis factor; VEGF, vascular endothelial growth factor.
